# Base Excision Repair in Physiology and Pathology of the Central Nervous System

**DOI:** 10.3390/ijms131216172

**Published:** 2012-11-30

**Authors:** Matthias Bosshard, Enni Markkanen, Barbara van Loon

**Affiliations:** Institute for Veterinary Biochemistry and Molecular Biology, University of Zürich-Irchel, Winterthurerstrasse 190, 8057 Zürich, Switzerland; E-Mails: matthias.bosshard@vetbio.uzh.ch (M.B.); enni@vetbio.uzh.ch (E.M.)

**Keywords:** brain, neurodegeneration, reactive oxygen species, DNA damage, base excision repair

## Abstract

Relatively low levels of antioxidant enzymes and high oxygen metabolism result in formation of numerous oxidized DNA lesions in the tissues of the central nervous system. Accumulation of damage in the DNA, due to continuous genotoxic stress, has been linked to both aging and the development of various neurodegenerative disorders. Different DNA repair pathways have evolved to successfully act on damaged DNA and prevent genomic instability. The predominant and essential DNA repair pathway for the removal of small DNA base lesions is base excision repair (BER). In this review we will discuss the current knowledge on the involvement of BER proteins in the maintenance of genetic stability in different brain regions and how changes in the levels of these proteins contribute to aging and the onset of neurodegenerative disorders.

## 1. Introduction

Our genome is under constant genotoxic stress. Endogenous agents, such as reactive oxygen species (ROS) produced during physiological cellular metabolism, have the potential to attack the DNA molecules, thus generating various oxidized lesions (reviewed in [1,2]). Additional sources of DNA damage represent exogenous insults, like ionizing radiation and UV-light exposure. As the maintenance of genomic integrity is of highest priority, living organisms have evolved multiple molecular mechanisms to repair damaged DNA. An inability to remove various types of DNA damages results in a broad spectrum of pathologies, such as neuronal deficits, immunodeficiencies, premature aging and cancer (reviewed in [3]).

**Figure 1:**
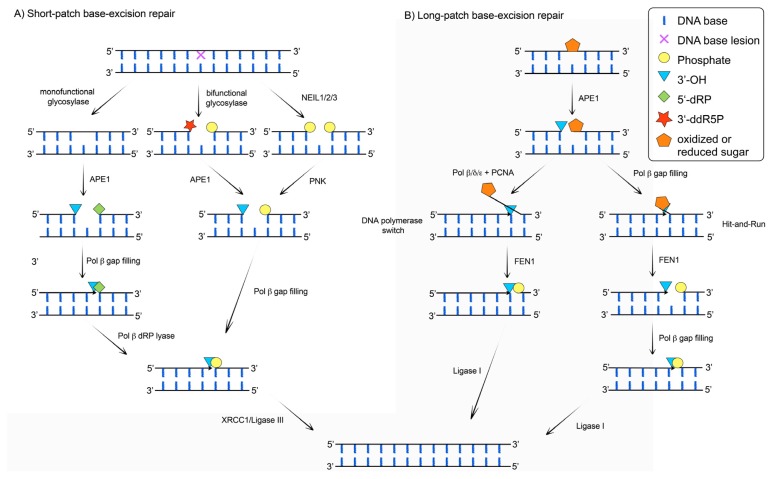
Short-patch (SP-) and long-patch base excision repair (LP-BER) sub-pathways. The damaged base is recognized and excised by a DNA glycosylase, resulting often in AP site formation, which is further processed by APE1. Subsequent end-processing generates 3′-OH and 5′-phosphate (5′-P) termini, enabling access of repair Pols. Depending on the number of newly incorporated nucleotides, the BER pathway divides into two sub-pathways: short-patch BER (SP-BER) and long-patch BER (LP-BER). (**A**) In SP-BER, a Pol β-mediated single nucleotide incorporation is followed by strand ligation, catalyzed by the XRCC1/DNA ligase III complex; (**B**) In contrast, LP-BER synthesizes a repair patch consisting of 2–12 nucleotides by: (i) the Hit-and-Run mechanism involving alternating FEN1 cleavage and Pols β synthesis; or (ii) the strand-displacement DNA synthesis concerted by Pols β and δ/ɛ. The 5′-flap, created during strand-displacement DNA synthesis, is removed by the FEN1 generating a nick. The FEN1 created nick is sealed by DNA ligase I. For more details see text.

Post-mitotic cells, like neurons of the central nervous system (CNS) possess a limited selection of the canonical DNA repair pathways, which makes them particularly sensitive to further DNA damage response (DDR) deficiencies [4]. A functional and efficient DDR is therefore of crucial importance to ensure their survival. Since neurons that are lost are generally not replaced, DNA repair is essential to sustain brain homeostasis. Given the high oxygen metabolism of the brain and the relatively low levels of antioxidant enzymes, ROS-induced oxidized DNA lesions represent a major type of neuronal DNA damage (reviewed in [5,6]). 7,8-dihydro-8-oxo-guanine (8-oxo-G) is one of the most frequently generated oxidized DNA lesions and thus often used as a marker of oxidative stress and ROS-damage. The predominant DNA repair pathway enabling efficient removal of small base damages due to ROS, alkylating agents or spontaneous decay, is base excision repair (BER) (Figure 1). This pathway is carried out through either short-patch (SP-) or long-patch BER (LP-BER) subpathways. The SP-BER (Figure 1A) is initiated by one of the eleven lesion-specific DNA glycosylases, grouped in three classes (monofunctional, bifunctional, endonuclease VIII (Nei)-like proteins (NEIL)) [7], which recognize and excise the damaged base from the DNA by hydrolyzing the *N*-glycosidic bond. In the case of monofunctional glycosylases (such as uracil *N*-glycosylase (UNG), single-strand-specific monofunctional uracil DNA glycosylase 1 (SMUG1), methyl-CpG binding domain protein (MBD4), thymine DNA glycosylase (TDG), MutY glycosylase homologue (MUTYH) and alkyladenine DNA glycosylase (AAG)), upon hydrolysis of the *N*-glycosidic bond an abasic (AP) site is generated and the DNA backbone is subsequently cleaved by the apurinic/apyrimidinic endonuclease 1 (APE1), producing a single nucleotide (1 nt) gap with 3′-hydroxyl (3′-OH) and a 5′-deoxyribose phosphate (5′-dRP) moiety, respectively. In addition to the *N*-glycosidic hydrolysis, bifunctional glycosylases (like 8-oxoguanine DNA glycosylase (OGG1) and Endonuclease III-like 1 (NTHL1)) cleave the phosphodiester backbone by β-elimination, through the AP lyase activity, giving rise to a 3′ terminal sugar phosphate (3′-ddR5P) and a 5′-phosphate residue. The 3′-ddR5P is further processed by APE1, resulting in a 1nt gap with 3′-OH terminus. In case the repair is initiated by NEIL1/2/3 glycosylases, after *N*-glycosidic hydrolysis, processing of the termini by β,δ-elimination is catalyzed, resulting in 3′- and 5′-phosphate residues, respectively. The 3′-phosphate is thereafter cleaved by polynucleotide kinase (PNK), producing a 1nt gap with 3′-OH terminus. In all situations the 1nt gap, created during SP-BER, will be filled by DNA polymerase (Pol) β through incorporation of one nucleotide. If the repair was initiated by monofunctional glycosylases, a polymerisation step is followed by Pol β 5′-dRP lyase activity. A newly synthetized product contains 3′-OH and 5′-phosphate termini that can be ligated by the X-ray repair cross complementing 1 protein (XRCC1)/DNA ligase III complex. If Pol β dRP lyase activity can not process the 5′ terminus created through glycosylase independent APE1 directed incision of natural AP sites (AP) or 2′-deoxyribonolactone (2′-dRL) residues, as well as oxidation or reduction of 5′-deoxyribose fragment, LP-BER subpathway (Figure 1B) will take place. Removal of such a blocking 5′ moiety can occur either through: (**i**) strand-displacement DNA synthesis where a switch to Pols δ or ɛ takes place after incorporation of first nucleotide by Pol β [8–11], or (**ii**) the Hit-and-Run mechanism by alternating flap endonuclease 1 (FEN1) cleavage and Pols β synthesis (reviewed in [2,12]). Extension by Pol δ/ɛ is mediated through displacement of the downstream strand, generating a 5′-flap that can be recognized and cleaved by FEN1. The resulting 3′-OH and 5′-phosphate termini are finally ligated by DNA ligase I.

Numerous studies in the past decades strongly associate the accumulation of DNA lesions, induced by DNA repair deficiencies, with a broad spectrum of progressive neurodegenerative disorders. Thus, the aim of this review is to summarize the current knowledge of the deficiencies in BER proteins associated with neurodegeneration and to illustrate the pivotal role of efficient oxidative DNA damage repair needed to protect the neurons of the CNS. We will present the findings gained from the various studies by following the proteins in the order they appear in the BER pathway (Table 1).

## 2. DNA Glycosylases

### 2.1. The Helix-Hairpin-Helix DNA Glycosylases

#### 2.1.1. OGG1

8-oxoguanine DNA glycosylase (OGG1) is a bifunctional DNA glycosylase that removes oxidized bases such as 8-oxo-G, 2,6-diamino-4-hydroxy-5-formamidopyrimidine (FaPyG) and 7,8-dihydro-8-oxoadenine (8-oxo-A) from the DNA ([13–22], and reviewed in [2]). Importantly, while OGG1 removes 8-oxo-G and FaPyG when base pared to a natural cytosine (C), 8-oxo-A will not be removed when paired to native thymine (T). OGG1 is expressed in at least four different splice forms in mammalian cells, of which at least two contribute differentially to BER pathways removing 8-oxo-G from nuclear (nDNA) and mitochondrial DNA (mtDNA) [23]. By catalyzing the excision of an oxidatively damaged base, OGG1 initiates a canonical SP-BER pathway that involves the action of APE1, Pol β and XRCC1/DNA ligase III to reconstitute the original intact base pair.

It is known that oxidative DNA damage plays a role in the process of ageing. OGG1 as one of the main regulators of 8-oxo-G levels in the genome, was shown to be widely expressed and active in human as well as rodent brains [24]. Initial transient decrease in OGG1 expression directly upon birth of mice, was followed by an increase after 8 weeks of age. Along this line, using a Comet-assay analysis of DNA damage in isolated neurons and astrocytes from the cortex of young (7 days), adult (6 months) and old (2 years) rats, Swain *et al.* revealed an age-dependent increase in the number of OGG1-sensitive sites, accompanied by a decrease in the OGG1 activity [25]. Further, by testing the activity of neuronal extracts from rat cerebral cortices in an *in vitro* assay with synthetic oligonucleotide duplexes, the authors observe a marked decline of 8-oxo-G repair capacity with age [26]. The decline could be attributed to a decrease in the expression levels of OGG1 and other BER enzymes including APE1 and Pol β. Supplementation of the neuronal extracts with the reduced components individually did not result in rescuing of the BER activity, suggesting that the age-dependent decline was not a result of an overall deficiency in the single DNA repair factors. However, addition of OGG1 together with Pol β and T4 DNA ligase markedly improved the BER activity and thus suggested that several BER proteins are limiting factors in adult and old neurons.

Acetylation of OGG1 has been shown to promote its enzymatic activity up to 10-fold *in vitro*[27]. Analysis of the OGG1 acetylation status in brain neurons of young rats revealed an increase in the acetylated form of OGG1 associated with either exercise or insulin-like growth factor-1 (IGF-1) treatment, a factor known to enhance neurogenesis [28]. In contrast, total OGG1 levels, as well as the amount of the acetylated OGG1 form, decreased with age in rats and correspondingly 8-oxo-G levels increased. The age-associated decrease in neurogenesis was possible to attenuate with exercise and IGF-1 treatment; at the same time exercise also improved the spatial memory, while IGF-1 treatment inhibited this process. These findings could potentially underline a role of oxidative DNA damage in age-related neuropathologies. Ogonovszky *et al.* further showed that neither were the levels of 8-oxo-G nor the OGG1 activity altered by exercise training in rats, suggesting that over-training does not induce oxidative stress in the brain and does not cause loss of memory [29].

Besides investigating the impact of the age-related decline on cellular level, studies of the mtDNA repair in particular revealed an association between DNA damage levels in the mitochondrial genome and different brain regions. By determining the mtDNA repair status in the central auditory system using a rat model of D-galactose-induced aging, Chen *et al.* observed a significant age-associated increase in mtDNA 4834 base pairs (bp) deletions and the number of terminal deoxynucleotidyl transferase–mediated uridine 5′-triphosphate-biotin nick end-labeling (TUNEL)-positive cells, a marker for apoptosis [30]. Interestingly, expression of Pol γ, the major mitochondrial Pol, and OGG1 were remarkably down-regulated in the auditory cortex. Thus, potentially indicating that during aging, increased mtDNA damage likely resulted from a decrease in its DNA repair capacity. These findings are supported by the work of Gredilla *et al.* addressing the efficiency of BER throughout the murine lifespan in mitochondria from cortex and hippocampus, both of which are regions severely affected during aging and in neurodegenerative diseases [31]. OGG1 activity peaked at middle-age in cortical mitochondria, followed by a significant drop at old age. However, only minor changes were observed in hippocampal mitochondria during the whole lifespan of the animals. Furthermore, OGG1 activity was lower in hippocampal than in cortical mitochondria. Taken together, these data suggest an important region-specific regulation of mitochondrial BER during aging.

The expression of OGG1 can also be modulated by many exogenous compounds, as shown by several studies discussed in the following. Cigarette smoke was found to induce DNA damage, as well as to alter OGG1 activity and distribution in several regions of the brain in neonatal mice; underlining the importance of cigarette smoke as risk factor for neurodevelopmental, as well as neurodegenerative disorders [32]. Fenvalerate is a synthetic pyrethroid widely used as pesticide in agriculture in developing countries and acts as neurotoxic compound in adults. To investigate the potential toxicity of fenvalerate to developing organisms, Gu *et al.* treated zebrafish larvae with this pesticide and found that OGG1 expression was down-regulated in a concentration-dependent manner. Fenvalerate also caused brain impairment during zebrafish development, further underlining the toxic nature of the compound especially during development [33]. Another pesticide, the organochlorine dieldrin, is a known neurotoxicant ubiquitously distributed in the environment and is toxic for dopaminergic neurons *in vitro*. Dieldrin slightly up-regulated OGG1 activity in proliferating PC12 cells, while the 8-oxo-G levels remained unchanged [34]. Differentiated PC12 cells on the other hand showed a longer lasting decline in OGG1 activity and a concomitant increase in 8-oxo-G levels. The differences between proliferating and differentiated cells might explain at least in part the vulnerability of post-mitotic neurons to oxidative stress and neurotoxins. A study that analyzed the impact of developmental exposure to lead (Pb), a known inducer of oxidative stress in the brain, found that cerebral 8-oxo-G levels were only transiently modulated early in life, at postnatal day 5, but were markedly elevated 20 months after the exposure had ceased [35]. OGG1 activity itself was not altered by developmental Pb exposure, resulting in loss of the age-dependent inverse correlation between OGG1 activity and 8-oxo-G accumulation. Exposure to Pb in old age did not have an impact on 8-oxo-G levels, suggesting that age-related oxidative damage accumulation and neurodegeneration could be markedly influenced by developmental disturbances.

Along the line of OGG1 involvement in development, OGG1 was found to be important for the determination of neural stem cell (NSC) differentiation by repairing mtDNA damage in differentiating neural cells [36]. NSCs derived from OGG1 knockout (ko) mice spontaneously accumulated mtDNA damage and shifted their differentiation direction toward an astrocytic lineage. A similar phenotype was observed when wild-type (wt) NSCs were subjected to mtDNA damaging insults, thus suggesting that mtDNA damage might be one of the primary signals for elevated astrogliosis and the lack of neurogenesis, a phenomenon observed after neuronal injury. Another study also demonstrated that small interfering RNA (siRNA) mediated knockdown (kd) of the DNA glycosylases OGG1 and endonuclease VIII (Nei)-like protein (NEIL) 3 decreased the differentiation ability of NSC, resulting in a decline of both neuronal and astrocytic gene expression after mitogen withdrawal, as well as a decrement in the stem cell marker Musashi-1 [37]. This suggests that OGG1 plays a role in governing essential NSC characteristics.

Besides the above-described impacts on development and ageing, alterations in OGG1 have been associated with numerous neurodegenerative disorders as elaborated bellow.

##### 2.1.1.1. Parkinson’s Disease

The primary cause of Parkinson’s disease (PD), the second most common age-related neurodegenerative disorder [38], is still unknown. However, the pathogenesis of PD has been linked to mitochondrial dysfunction and oxidative stress (reviewed in [39,40]). Both of these factors are regarded as important contributors to neuronal death in the substantia nigra (SN) of PD patient brains. Indeed, besides increased 8-oxo-G levels [41], it has been shown that, among several other DNA glycosylases, OGG1 is up-regulated in the SN of PD patients [42]. The potential involvement of OGG1 in PD was further supported through the finding that aged OGG1 ko mice developed an age-associated mild parkinsonian phenotype, which manifested among others in spontaneous locomotor behavior and decreased striatal dopamine levels [43]. Furthermore, this study showed that young OGG1 ko mice were more susceptible to the dopaminergic toxin 1-methyl-4-phenyl-1,2,3,6-tetrahydropyridine than their wt littermates. Finally, an age-associated increase in 8-oxo-G levels was seen in this mouse model, further validating this mouse strain as a possible model for PD. Nakabeppu *et al.* showed a significant increase in 8-oxo-G in mtDNA as well as an elevated expression of 8-oxo-G dGTPase (MTH1), OGG1 and MutY glycosylase homologue (MUTYH) in nigrostriatal dopaminergic neurons of PD patients, suggesting that the buildup of oxidized DNA lesions may be involved in the loss of dopaminergic neurons [44]. Furthermore, MTH1-null mice, exhibiting an increased accumulation of 8-oxo-G in striatal mtDNA, displayed a more extreme neuronal dysfunction after 1-methyl-4-phenyl-1,2,3,6-tetrahydropyridine administration than wt mice; potentially indicating that oxidative DNA damage presents a major risk factor for PD.

The OGG1 S326C polymorphism is commonly associated with an increased risk for various kinds of cancer, such as lung [45] and breast cancers [46]. Coppedè *et al.* investigated whether the occurrence of PD correlates with the OGG1 S326C polymorphism by screening 139 sporadic PD patients and 211 healthy matched controls [47]. Neither did the allele frequency of C326 differ between the groups (0.20 in PD patients and 0.19 in controls; *p* = 0.817), nor did the differences in genotype frequencies. Furthermore, there was no association of S326C with the disease age at onset (*p* = 0.791). Overall, these results suggested that the OGG1 S326C polymorphism is not associated with sporadic PD.

Taken together, OGG1 seems to have a crucial influence on the pathogenesis of PD, but more studies are needed to shed light on the exact mechanism connecting oxidative DNA damage and PD.

##### 2.1.1.2. Amyotrophic Lateral Sclerosis

Amyotrophic lateral sclerosis (ALS) is the most common motor neuron disease with adult onset (reviewed in [48]). It is characterized by progressive degeneration of motor neurons in the anterior horn cells of the spinal cord, the brain stem and the cerebral cortex. Increased levels of 8-oxo-G have been found in the spinal cords of ALS patients [49] and further evidence hints at a deficiency in mtDNA repair underlying the pathogenesis of ALS (reviewed in [39]). A study analyzing the association of the OGG1 S326C polymorphism in sporadic ALS found that both the C326 allele (*p* = 0.02) and the combined S326C + C326C genotype (OR = 1.65, 95% CI = 1.06–2.88) increased the risk of ALS [50]. Even though the risk was higher, no significant association between the disease phenotype and the S326C polymorphism, with respect to the age, onset site, as well as disease progression, could be observed. These results suggested a possible involvement of the human OGG1 S326C polymorphism in the pathogenesis of sporadic ALS.

The Cu/Zn-superoxide dismutase 1 (SOD1) is an antioxidant enzyme that converts superoxide anions (O_2_^−^) to hydrogen peroxide (H_2_O_2_) and thus contributes to the control of the oxidative DNA damage levels. Murakami *et al.* used transgenic mice carrying mutant SOD1 as an animal ALS model to analyze the expression of OGG1 [51]. They found that the nuclear form of OGG1 was up-regulated in presymptomatic mice, while mitochondrial OGG1 levels remained stable, thus potentially indicating that the de-regulation of protective mechanisms against oxidative stress could contribute to ALS.

##### 2.1.1.3. Triplet Repeat Expansion Diseases

There are at least 18 different neurological diseases, among them Huntington’s disease (HD) and several inherited ataxias, that have been linked to the expansion of trinucleotide repeats (TNRs) in the human genome (reviewed in [52]). In HD, CAG triplet expansion occurs in the Huntingtin gene in post-mitotic neurons and results in altered interaction of the Huntingtin protein with other binding partners (reviewed in [39]). OGG1 has been suggested to play a role in this pathogenesis through the initiation of the BER pathway to excise 8-oxo-G present in these tracts [53]. This study suggested a “toxic oxidation” model in non-dividing cells by which the OGG1-initiated repair of 8-oxo-G triggers an iterative oxidation-excision cycle that culminates in progressive age-dependent expansion of the CAG repeats. The model predicts that triplet repeat expansion results from error-prone repair steps downstream of OGG1 and APE1 action, namely by strand displacement/slippage of the Pol during gap-filling reaction. Such slippage results in the formation of a hairpin structure, which gets stabilized by MSH2/MSH3, and cannot be recognized by FEN1 for flap trimming, since the 5′ end of the flap is hidden in the hairpin structure. Importantly, this somatic age-dependent expansion is independent of cell division, as it takes place in terminally differentiated cells. Maybe somewhat surprisingly the largest expansions in TNRs occur in non-dividing tissues, where besides the BER-mediated toxic oxidation also nucleotide excision repair (NER) pathway has been implicated in pathogenesis of TNR expansions (reviewed in [54]). Several models have also been proposed to account for the TNR expansion in dividing tissue [54]. Interestingly, Jarem *et al.* showed that, while OGG1 activity is comparable on duplexes (*i.e.*, linear dsDNA molecules) containing either TNR or a mixed sequence, OGG1 shows a reduced affinity and excision activity for 8-oxo-G in hairpin substrates [55]. These findings suggest that 8-oxo-G accumulates at hairpin structures, which can subsequently be incorporated into duplexes, thus giving rise to a TNR expansion that still contain unrepaired 8-oxo-G lesions capable of starting yet another toxic cycle of expansion. In contrast to Kovtun *et al.* a study by Lin *et al.* showed that the kd of OGG1 and APE1 did not affect repeat instability [56]. This discrepancy to the above mentioned data might well be a result of cell-line specific effects. Interestingly, CAG repeat expansion in HD is targeted preferentially to the striatum, while other brain regions, such as the cerebellum, remain spared. Investigating this phenomenon, Goula *et al.* found that oxidative DNA damage abnormally accumulates at CAG repeats in a length-dependent, as well as age- and tissue-independent manner in HD mice [57]. Analysis of protein levels and enzymatic activities in the striatum and cerebellum of HD mice, showed a striatum-specific down-regulation of proteins acting in the BER pathway downstream of OGG1, correlating with increased somatic CAG instability in the striatum over the cerebellum in HD mice. This suggests that the relative levels of BER proteins in different tissues potentially contribute to the disease manifestation. Besides wt OGG1, a recent study investigated the influence of the OGG1 S326C polymorphism on HD [58]. Both mono- or biallelic bearers of the mutant S326C allele tended to have an increased number of CAG repeats within the expanded HD allele (*p* = 0.049). Furthermore, mainly heterozygous subjects showed a significant (*p* = 0.041) earlier disease onset than OGG1 wt individuals, suggesting a possible role of the human OGG1 S326C polymorphism in the development of HD.

##### 2.1.1.4. Stroke/Ischemia/Hypoxia

Stroke is the third leading cause of death worldwide and its prevalence is steadily increasing (reviewed in [59]). It is mainly caused by thrombosis, embolism or hypotension and leads to a reduction of the blood flow insufficient to sustain normal cellular function (ischemia). Since the brain is an organ that consumes a large amount of oxygen, it is considered to be exposed to increased levels of oxidative DNA damage. The capacity to repair the oxidized DNA lesions is regarded as an important factor that determines neuronal survival after an ischemic insult (reviewed in [60]). Several studies have investigated means by which levels of OGG1 are regulated in different neuronal tissues upon ischemia and reperfusion. The results are quite heterogeneous and seem to depend largely on the model system used and analyzed. A study done by He *et al.* investigated the role of oxidative DNA damage in secondary remote tissue damage within the ventroposterior nucleus (VPN) after distal middle cerebral artery (MCA) occlusion in hypertensive rats [61]. Immunohistochemical analysis of the ipsilateral VPN revealed an increase in 8-oxo-G, while OGG1 immunoreactivity significantly decreased two weeks after cortical infarction (all *p* < 0.01). These findings, together with the notion that ebselen, a glutathione peroxidase mimic, significantly attenuated the loss of neurons and counteracted the effects in 8-oxo-G and OGG1, suggest a potential involvement of oxidative DNA damage in ischemia-induced delayed neuronal death within the VPN region. To understand the impact of oxygen and glucose deprivation on BER in two different regions of the hippocampus (CA1 and CA3/fascia dentata), Rolseth *et al.* measured the enzyme activities and gene expression levels of DNA glycosylases and AP-endonucleases in organotypic rat hippocampal slice cultures [62]. They found that under basal conditions AP-endonuclease activity and base removal of 1,*N*^6^-ethenoadenine (ɛA) and 8-oxo-G were approximately 20%–35% higher in the CA3/fascia dentata than in the CA1 region. In contrast to the AP-endonuclease activity and ɛA base removal, 8-oxo-G excision did not significantly change after 30 min or 8 h of oxygen and glucose deprivation. Additionally, reverse transcription-quantitative polymerase chain reaction (RT-qPCR) showed no changes in the transcription of OGG1 or any other of the investigated DNA glycosylases in response to a treatment of 30 min. The study however did not investigate transcriptional levels at later time points. The authors concluded that the relatively low capacity for BER under basal conditions and the apparent failure to up-regulate the repair of oxidative damage after oxygen and glucose deprivation might contribute to the high vulnerability of the hippocampal CA1 region to ischemic injuries.

A brief period of sublethal preconditioning ischemia can attenuate the injury extent arising from subsequent severe ischemia, possibly involving the activation of a variety of pathways that promote neuronal survival. Li *et al.* investigated whether BER could be induced as endogenous adaptive response, preventing the detrimental effect of oxidation damage, in a rat model where several episodes of ischemic preconditioning were applied prior to MCA occlusion to mimic a stroke [63]. In this study, ischemic preconditioning markedly reduced the nuclear accumulation of 8-oxo-G and other oxidized DNA lesions, leading to a decreased DNA damage response measured by p53 activation and nicotinamide adenine dinucleotide (NAD) depletion. Furthermore, measurements of BER activities in nuclear extracts revealed that Pol β-mediated BER was markedly increased after ischemic preconditioning, likely as a result of an increase in the expression of Pol β, APE1 and OGG1 [63]. These results suggest that the protective effects of ischemic preconditioning might be partly due to enhanced repair of endogenous oxidized DNA lesions. Subsequent analysis of OGG1 ko mice revealed that OGG1 protects neurons against ischemia-induced oxidative DNA damage, as measured by accumulation of 8-oxo-G in the brain, and changes in cell death levels [64]. In contrast to the findings of Rolseth *et al.*, this study showed an ischemia-induced elevation of 8-oxo-G incision activity resulting from an increase in the levels of a nuclear OGG1 isoform, peaking at around 6 h post treatment, thereby suggesting an adaptive response to oxidative nuclear DNA damage. From this it seems that OGG1 plays a role in reducing brain damage and improving functional outcome after ischemia by repairing oxidatively damaged nuclear DNA. Ischemia/reperfusion has been shown to lead to elevated matrix metalloproteinase activity, which further promoted (i) the degradation of the two important DNA repair proteins poly-ADP ribose polymerase 1 (PARP1) and XRCC1 and (ii) the accumulation of oxidative DNA damage after an ischemic stroke [65]. Concomitantly, analysis of primary cortical neurons subjected to oxygen-glucose deprivation displayed a marked decrease in OGG1, among other BER proteins [66]. Thus, it seems that the intranuclear gelatinase activity of matrix metalloproteinases acts in an intrinsic apoptotic pathway that is activated as a response to DNA damage in neurons during acute stroke injury.

Hypothesizing that ischemia-reperfusion injuries in the spinal cord caused 8-oxo-G production and thus activated the DNA repair system involving OGG1, Lin *et al.* analyzed the spinal cords of rabbits after infrarenal aortic occlusion from 1 h to 48 h of reperfusion [67]. The results demonstrated that 8-oxo-G was present in the grey matter after reperfusion and that, among other DNA repair proteins, the levels of OGG1 were markedly increased, peaking at around 6 h after reperfusion. Therefore, it seems that DNA repair proteins are rapidly expressed after spinal cord ischemia and subsequent reperfusion.

Hyperoxic reoxygenation of asphyxiated newborns could cause increased damage to DNA. To investigate this matter, and also to test whether therapeutic hypothermia might attenuate the development of brain damage after asphyxia, newborn pigs were subjected to hypoxia followed by either normothermia or total body cooling [68]. 8-oxo-G was found to be elevated in the urine of hypoxic pigs, but these levels were not affected by hyperoxia or hypothermia. 8-oxo-G levels in brain and liver tissue did not change after any treatment. OGG1 expression in the hippocampus and the liver was down-regulated by hypothermia, without influencing the accumulation of oxidative DNA damage in genomic DNA. Also expression of OGG1 in the brain was not affected by hyperoxia. Thus, this study confirmed an increase in oxidative stress after hypoxia. In addition, DNA repair glycosylases were shown to be down-regulated by hypothermia but this had no effect on the accumulation of oxidative damage in genomic DNA.

##### 2.1.1.5. Alzheimer’s Disease

Alzheimer’s disease (AD) is a progressive neurodegenerative disease characterized by memory impairment, cognitive decline and behavioral changes. As such, it is the most common age-associated severe dementia. Molecular mechanisms that lead to AD are slowly being unveiled and include the deposition of amyloid β-peptide (Aβ) plaques as well as accumulation of oxidized base damage both in the nuclear as well as the mtDNA (reviewed in [39]). Still, the involvement of DNA repair in the pathogenesis of AD is far from being completely understood.

An increase in oxidative DNA damage and a concomitant reduction in OGG1 mediated BER were detected in vulnerable brain regions in various stages of AD (reviewed in [39]). A study investigating, whether oxidative DNA damage is already present in a recently described preclinical stage of AD showed a significant increase in 8-oxo-G levels as well as elevated OGG1 protein levels in the hippocampus and the parahippocampal gyri [69]. Furthermore, an increase in OGG1 mRNA was measured in the superior and middle temporal gyri. Summarizing, these data suggested that oxidative damage to DNA induced a compensatory increase in OGG1 expression early in the pathogenesis of AD.

Feng *et al.* showed that Aβ induces oxidative DNA damage in murine brains, and that this effect can be counteracted by soybean isoflavones (SIFs), previously found to exhibit neuroprotective effects by suppression of oxidative stress [70]. Mechanistically, mRNA and protein levels of OGG1 were up-regulated by SIFs, suggesting that the protective effects of SIF might be at least partly associated with the regulation of oxidative DNA damage repair by OGG1. In a model system of AD using rabbits fed with a cholesterol-rich diet, it was shown that 8-oxo-G accumulated in the brain, primarily in the hippocampus, and induced a range of DNA repair activities [71]. In the same study, OGG1 was found to physically interact with the xeroderma pigmentosum group B-complementing protein (XPB), which may potentially account for a mechanism involving these DNA repair responses. Furthermore, in contrast to wt mice, mice lacking OGG1 showed no interleukin-6 (IL-6) activation but a drastic increase of the pro-inflammatory cytokine tumor necrosis factor-α (TNF-α), suggesting that OGG1 may be involved in cytokine production induced by high cholesterol levels, and thus affecting neurodegeneration.

##### 2.1.1.6. Involvement of OGG1 in other Neurodegenerative Disorders

Depression is known to induce elevated oxidative stress levels in peripheral blood of affected patients [72]. However, a study by Teyssier *et al.* found no significant impact of depression on the expression of OGG1 and several other oxidative stress-response proteins in the prefrontal cortex [73]. They concluded that the pathogenic role of oxidative stress in the brain could thus not be inferred from the alteration of peripheral parameters. However, as many other studies have shown that the amount of 8-oxo-G levels do not necessarily correlate with the levels of OGG1 (and other related proteins), it might have been interesting to measure the actual 8-oxo-G content in the brain in this study as well.

Mitochondrial OGG1 was down-regulated both at mRNA and protein levels in a pilocarpine-induced status epilepticus in the hippocampi of male rats, suggesting that lowering of mitochondrial BER enzymes may aggravate mtDNA damage and mitochondrial deficiency after the onset of a status epilepticus [74].

Cockayne syndrome (CS) is a rare recessive childhood-onset neurodegenerative disease, characterized by a deficiency in the DNA repair pathway of transcription-coupled NER (TC-NER). Mice with a targeted deletion of the *CSB* gene are used as a model for this disease. It was found that a double kd of CSB and OGG1 did not enhance the neurodegenerative phenotype, suggesting that in this disease unrepaired endogenous lesions are mostly substrate for NER, but not BER [75].

In summary, OGG1 is an enzyme that has been widely implicated to play a role in various physiological states of the brain and neuronal tissue, and its function is correlated to the onset of many neurodegenerative diseases. Still, the exact mechanisms that lead to the respective disorders, as well as the reasons why the regulation of OGG1 in different brain regions is so divergent, are far from being understood. Further studies are needed to unequivocally clarify the precise role of OGG1 in neurodegeneration.

#### 2.1.2. MUTYH

MUTYH (also sometimes called MUTY or MYH) is, like OGG1, a DNA glycosylase of the helix-hairpin-helix (HhH) family. It mediates the removal of adenine (A) paired with an 8-oxo-G [76,77], a situation that arises when replicative Pols bypass 8-oxo-G in an inaccurate manner by inserting a wrong A instead of a correct C. With this action MUTYH gives rise to a novel BER pathway involving Pol λ that reconstitutes the correct C:8-oxo-G base pair, which is then a substrate for OGG1 ([78–80], and reviewed in [2]); Several nuclear as well as mitochondrial isoforms of MUTYH are present in mammalian cells [81]. Biallelic mutations in MUTYH predispose to a familial adenomatous polyposis variant called MUTYH-associated polyposis (MAP) [82]. However, no evidence of increased risk for cancers of the brain tissue has been found in MAP patients [83].

Up to date, a limited number of insights have been obtained regarding the potential roles of MUTYH in the brain. A study using MUTYH ko mice showed that there was no time-dependent accumulation of 8-oxo-G in brain tissue [84]. Interestingly, Lee *et al.* showed that levels of one embryonic isoform of MUTYH could be detected in rat brains at the E14 embryonic stage, after which it decreased during embryonic and neonatal development, while new isoforms appeared and gradually increased in the neonate and adult brain [85]. It seemed that during embryonic development expression levels of MUTYH followed the expression profile of proliferating cell nuclear antigen (PCNA). In addition, these proteins also colocalized in the nucleus. At later time points, when the levels of PCNA declined, MUTYH was detected primarily outside the nucleus. An activity for excision of A opposite 8-oxo-G was detected in all the extracts. Even though the authors suggested that MUTYH might be primarily involved in post-replicative repair of nDNA, it is possible that MUTYH might rather be involved in repair of mtDNA in post-mitotic neurons.

Though not much is known about the detailed role of MUTYH in the brain DDR, alterations in the MUTYH homeostasis have been associated with various neurodegenerative diseases, such as PD.

##### 2.1.2.1. Parkinson’s Disease

Analogous to OGG1 levels, Fukae *et al.* demonstrated in the same study that also the levels of MUTYH are up-regulated in the SN of PD patients, suggesting that MUTYH is involved in the maintenance of mtDNA in PD brain [42]. As mentioned earlier, Nakabeppu *et al.* were able to show a significant increase in 8-oxo-G in mtDNA as well as an elevation in expression of MTH1, OGG1, and MUTYH in nigrostriatal dopaminergic neurons of PD patients, suggesting that the accumulation of these lesions may be involved in the loss of dopaminergic neurons [44]. Following the same line, Arai *et al.* found by immunohistochemical and biochemical analysis that MUTYH was up-regulated in the mitochondria of the SN of PD patients [86]. Western blot analysis identified a 47 kDa molecule as the major isoform in these brains. Surprisingly, this isoform was localized to the mitochondria and stemmed from the alpha4 mRNA, even though it lacks the mitochondrial targeting sequence.

##### 2.1.2.2. Stroke/Ischemia/Hypoxia

Similarly to the OGG1 study [61], He *et al.* investigated the impact of oxidative DNA damage in the secondary remote tissue damage, within the VPN after distal MCA occlusion, in hypertensive rats with respect to the MUTYH expression [87]. Immunohistochemical imaging analysis showed a distinct nuclear and cytoplasmic distribution of MUTYH in the entire region of the VPN. Compared with the sham group, the number of MUTYH positive cells decreased upon surgery. Additionally, treatment with ebselen was able to significantly increase the levels of MUTYH compared to the controls. In summary, a marked decrease of MUTYH in the VPN after 2 weeks of MCA occlusion was observed, and this effect could be counteracted by ebselen.

The same study that showed an increase of OGG1 levels after ischemia-reperfusion injuries in the spinal cord also demonstrated an increase of MUTYH levels after the treatment [67]. This suggested that indeed MUTYH levels could be up-regulated in response to spinal cord ischemia and subsequent reperfusion. Similarly, Lee *et al.* demonstrated a strong increase in MUTYH mRNA and protein levels upon respiratory hypoxia, accompanied by the formation of 8-oxo-G *in vivo* in rat brains [88]. *In situ* hybridization analysis revealed expression patterns of MUTYH mRNA in hippocampal, cortical and cerebellar regions. The same group demonstrated, that MUTYH is abundantly expressed in the rat brain, with isoforms that were exclusive to brain tissue and localized to neuronal mitochondria [89]. In addition, removal of 8-oxo-G induced by hypoxia was accompanied by a spatial increase in MUTYH immunoreactivity, as well as an increase in of one of the three mitochondrial MUTYH isoforms. Taken together, this suggested the existence of inducible and non-inducible MUTYH isoforms in the brain. Also, CoCl_2_, an agent that mimics hypoxia and induces oxidation damage, was found to induce damage to mtDNA, but not to nDNA, in rat neuronal PC12 cells [90]. This finding coincided with an elevation of MUTYH protein levels, further underlining the idea that mtDNA repair processes involving MUTYH can be induced by the presence of mtDNA damage.

##### 2.1.2.3. Involvement of MUTYH in Other Neurodegenerative Disorders

Examining changes in the levels of selected DNA repair enzymes and mtDNA damage in retinas from the eyes of young and old rodents, Wang *et al.* found an age-dependent increase in 8-oxo-G that co-localized with the mitochondrial enzyme superoxide dismutase, suggesting damage to mtDNA primarily in photoreceptors and retinal ganglions [91]. The expression levels of MUTYH seemingly decreased with age, consistent with the idea that an age-related increase in mtDNA damage is likely due to a decreased repair capacity in aged retinas and thus may contribute to age-related retinal diseases.

Equine Cerebellar Abiotrophy (CA) is a neurological disease found in Arabian horses caused by post-natal degeneration of the Purkinje cells of the cerebellum. A linkage analysis discovered that CA-affected horses display reduced expression of MUTYH due to a single nucleotide polymorphism (SNP) approximately 1200 bp upstream of the MUTYH gene, which is adjacent to a possible site for the transcription factor GATA2 [92]. The authors suggested that this SNP might have a regulatory effect on MUTYH by negatively affecting the affinity of GATA2 and thus contributing to the onset of CA.

Taken together, the evidences point at a potentially important role of MUTYH in the pathogenesis of neurodegenerative diseases such as PD and stroke, as well as age-dependent retinal degeneration and equine cerebellar abiotrophy. Given the importance of OGG1 in oxidation damage disorders, it is not surprising that MUTYH may also be implicated in some of these diseases, as it acts in a pathway that is very much depending on OGG1. It would be interesting to see whether also the entire pathway for correction of A: 8-oxo-G mismatches downstream of MUTYH, involving Pol λ and some of the LP-BER components, is of similar importance.

#### 2.1.3. MBD4

The methyl-CpG binding domain protein MBD4 (also known as MED1) is a DNA glycosylase that belongs to the MBD protein family within the HhH domain superfamily. It processes a wide substrate range of DNA base lesions mispaired with guanine (G), such as uracil (U), 5-fluorouracil (5-FU), 3,*N*^4^-ethenocytosine (ɛC) and T ([93–95], and reviewed in [7]), Mbd4 ko mice are viable and show no developmental defects [96,97]. Though lack of MBD4 does not lead to defects in mice, it has been found that MBD4 mRNA levels are significantly up-regulated in the hippocampus of both schizophrenia and bipolar disorders suggesting a potential involvement of this glycosylase in human neurodegenerative diseases [98]. To understand the exact mechanism how MBD4 contributes to these disorders, future studies are needed.

#### 2.1.4. NTHL1

Endonuclease III-like 1 (NTHL1, also known as NTH1) is a DNA glycosylase that belongs to the family of endonuclease III-like 1 proteins, a subfamily of HhH DNA glycosylases. It catalyzes excision of ring fragmented purines or oxidized pyrimidines like thymine glycol (Tg), 4,6-diamino-5-formamidopyrimidine (FaPyA), FaPyG, 5-hydroxycytosine (5-OHC) and 5-hydroxyuracil (5-OHU) when paired to G in double stranded DNA ([99–106], and reviewed in [7]). It is assumed that loss of NTHL1 function can be compensated for by NEIL glycosylases, because NTHL1 ko mice show no abnormalities [107,108] As was the case for NEIL1 and NEIL2, also no association for NTHL1 with the risk of developing multiple sclerosis was found [109]. So far, nothing more regarding a possible role of NTHL1 in neurodegenerative diseases is known.

### 2.2. The Endonuclease VIII-Like Glycosylases

#### 2.2.1. NEIL1

NEIL1 is a DNA glycosylase that belongs to the family of endonuclease VIII (Nei)-like proteins. Its preferred substrates are damaged pyrimidines and purines, such as Tg, FaPyA, FaPyG and others, but also 8-oxo-G and 5-OHU in double stranded DNA and bubble structures ([102,103,105,108,110–119], and reviewed in [7,120]), NEIL1 ko mice display a phenotype very close to the metabolic syndrome, and harbor increased levels of DNA damage in their mtDNA [121]. NEIL1 mRNA has been detected in different mammalian tissues including the brain and both its mRNA and protein levels were shown to increase during S-phase [102]. Further, widespread NEIL1 expression was reported at all ages in mice and it even increased with age, as did FaPyG lesion (induced by treatment of DNA with *N*-[^3^H]methyl-*N*′-nitrosourea) excision activity in all brain regions tested [24]. By investigating the efficiency of mitochondrial BER during the murine lifespan in the cortex and hippocampus, Gredilla *et al.* found that, similarly to OGG1; NEIL1 activity reached its maximum at middle-age in cortical mitochondria followed by a significant drop at old age, while only minor changes were observed in hippocampal mitochondria [31]. In addition, NEIL1 DNA glycosylase activity was lower in hippocampal than in cortical mitochondria. These findings indicate that regulation of mitochondrial NEIL1 activity in the brain is region and age specific.

Among other BER enzymes, Rolseth *et al.* investigated the impact of OGD on NEIL1 activity and gene expression levels in organotypic rat hippocampal slice cultures (particularly in the regions CA1 and CA3/fascia dentata) [62]. While base removal of U did not differ between the two hippocampal regions, removal of 5-OHU was slightly less efficient in CA3/FD than in CA1. After 30 min of OGD an increase in the activity on ɛA by approximately 25% could be detected in CA1, whereas activities for 8-oxo-G, 5-OHU and U remained unchanged. Later, 8 h after OGD, none of the enzyme activities differed from control values. As for OGG1, transcription of NEIL1 was not changed in response to OGD treatment at time point 0 h.

Englander *et al.* measured the expression and activities of BER enzymes during brain development where the physiological transition of neuronal cells from the proliferative to the post-mitotic differentiated state takes place [122]. Expression of NEIL1 increased during brain development concomitant with maintenance of the capacity for excision of 5-OHU from bubble structured DNA in the mature rat brain, suggesting a potential role of NEIL1 in the maintenance of the integrity of transcribed DNA in the post-mitotic brain. A recent study by Canugovi *et al.* demonstrates that NEIL1 ko mice exhibit an impairment in memory retention, as assessed by a water maze test [123]. However, these mice did not display abnormalities in motor performance, anxiety or fear conditioning. Furthermore, the deficiency in NEIL1 results in an increase in brain damage after ischemia/reperfusion due to apoptosis. Also, in these mice the incision activity of 5-OHU in a bubble structure was lower in the ipsilateral sides of ischemic brains as well as in mitochondrial lysates of unstressed old ko mice, suggesting that NEIL1 is a central player in learning, memory and neuronal protection against ischemia.

##### 2.2.1.1. Involvement of NEIL1 in Neurodegenerative Disorders

Several studies have addressed the importance of NEIL1 in neurological conditions, such as CS, multiple sclerosis and depression. Hypersensitivity of CSB-deficient cells to oxidative stress hint to a defect in oxidative DNA damage repair contributing to the phenotype. A study that examined the role of CSB in the repair of FaPyG and FaPyA, both substrates for NEIL1, found that CSB ko mice have a higher level of endogenous FaPyG and FaPyA in nDNA from brain, compared to wt mice [115]. Furthermore, CSB was co-immunoprecipitated and co-localized with NEIL1 in HeLa cells and stimulated NEIL1 activity *in vitro*. Depletion of CSB and NEIL1 from HeLa cells by short hairpin RNA (shRNA) strongly inhibited the repair of induced FaPyG, suggesting that CSB plays a role in repair of FaPyG lesions, possibly through the interaction with NEIL1. Further, these findings implicate that FaPyG and FaPyA lesions and thus NEIL1 may have a causal role in the pathogenesis of CS.

No association of NEIL1 with the risk of developing multiple sclerosis could be found [109]. Similarly, the study by Teyssier *et al.*, besides no significant changes in the OGG1 expression, did not detect an impact on NEIL1 levels in the prefrontal cortices of the patients suffering from depression [73]. Using the HD transgenic R6/1 mouse model, very recently Mollersen *et al.* demonstrated that the deletion of exon 2 of NEIL1 in mice leads to a significant reduction in somatic TNR expansions, when compared to their NEIL1 wt littermates [124]. Interestingly, while it could also be detected in female mice, the reduction of somatic expansions was more pronounced in male mice. Additionally, the authors found that NEIL1 binds and excises 5-OHC much more efficiently in duplex DNA than in hairpin substrates, suggesting that NEIL1 initiated BER of cytosine-derived oxidized lesions could be involved in the initiation of TNR expansions, additionally to other DNA modifications.

##### 2.2.1.2. Stroke/Ischemia/Hypoxia

As already mentioned, NEIL1 has been linked to changes in oxygen levels and pathological conditions such as ischemia and stroke [123]. By addressing the effect of hyperoxic reoxygenation and therapeutic hypothermia on the development of brain damage after asphyxia in newborn pigs, as described in the OGG1 subchapter, transcription of NEIL1 was significantly down-regulated in the hippocampus, cortex, striatum and liver upon hypothermia, [68]. However, no effect on the accumulation of oxidative DNA damage in genomic DNA could be visualized. Like OGG1, NEIL1 expression in the brain was unaffected by hyperoxia. Thus, even though NEIL1 was down-regulated by hypothermia, this had no effect on the accumulation of oxidative damage in genomic DNA.

In conclusion, NEIL1 seems to be important for the development of the brain, memory and learning, as well as in response to stroke and ischemia and it has been implicated in CS. What the exact roles of NEIL1 in the different parts of the brain are still remains unresolved and will be the subject of future studies.

#### 2.2.2. NEIL2

Like NEIL1, NEIL2 belongs to the family of endonuclease VIII (Nei)-like proteins. Its preferred substrates are strongly overlapping with the ones of NEIL1 and include oxidized pyrimidines, such as Tg, 5-OHU, 5-OHC, 5,6 dihydrothymine and 5,6 dihydrouracil in double stranded DNA and bubble structures ([111,119,125], and reviewed in [7,120]). NEIL2 mRNA has been detected in the brain, but unlike NEIL1, the expression of NEIL2 was independent of the cell cycle stage [111]. Analysis of the distribution patterns in mouse brains showed widespread expression of NEIL2 at all ages, and the excision activity of chemically induced FaPyG lesions increased with age in all brain regions tested [24].

Rolseth *et al.* found that transcription of NEIL2 in two different regions of the hippocampus was not changed in response to OGD treatment at time point 0 h [62]. As was the case for NEIL1, expression of NEIL2 levels increased during the physiological transition of neuronal cells from the proliferative to the post-mitotic differentiated state in brain development [122]. This was concomitant with the maintenance of the capacity for excision of 5-OHU from bubble structured DNA in the mature rat brain, suggesting a role for NEIL1 and NEIL2 in the maintenance of the integrity of transcribed DNA in the post-mitotic brain. Similarly to NEIL1, no association between NEIL2 and the risk of developing multiple sclerosis was found [109]. Future studies are needed to completely understand if and how NEIL2 could be associated with different neurodegenerative diseases.

#### 2.2.3. NEIL3

Similarly to NEIL1 and NEIL2, NEIL3 also belongs to the family of endonuclease VIII (Nei)-like proteins. In contrast to the two former glycosylases, NEIL3 excises FaPyG and FaPyA lesions but is inactive on 8-oxo-G ([126,127] and reviewed in [7]). Additionally, the mouse ortholog was shown to remove a broad spectrum of DNA base lesions on single-stranded DNA substrates, including secondary oxidation products of 8-oxo-G, such as spiroiminodihydantoin and guanidinohydantoin, suggesting that NEIL3 prevents the accumulation of these cytotoxic and mutagenic lesions in mammalian cells [127]. Though NEIL3 ko mice are viable and fertile, NEIL3 has been implicated to play a role in hematopoiesis or the immune system, since it is preferentially expressed in hematopoietic tissues [128]. In brains of newborn mice, NEIL3 revealed a discrete expression pattern in the subventricular zone, the rostral migratory stream, and the hilar region of the hippocampal formation, all of which are brain regions known to harbor stem cell populations [24]. Expression of NEIL3 decreased with age, and in brains of old mice it could be only detected in layer V of the neocortex. The distribution of NEIL3 thus indicates a potentially specific role of this enzyme in stem cell differentiation. Along with this study, expression pattern analysis of NEIL3 in the brain during mouse embryonic development revealed a tight regulation at both temporal and spatial levels. High expression of NEIL3 was observed at embryonic days 12–13, which coincides with the start of neurogenesis [129]. Subsequently, the expression of NEIL3 decreased gradually, and it could not be detected anymore in adult brains by RT-qPCR. Interestingly, expression during embryogenesis and in newborn mice was observed in areas with neural stem and progenitor cells, such as the subventricular zone and the dentate gyrus, suggesting that brain areas with neurogenesis and a high proliferative potential specifically express NEIL3. Subsequently, Sejersted *et al.* demonstrated a profound neuropathology in NEIL3 ko mice, which was characterized by a reduced number of microglia and a loss of proliferating neuronal progenitors in the striatum after hypoxia-ischemia [130]. Furthermore, NEIL3 ko neural stem/progenitor cells displayed an inability to increase neurogenesis and a reduced capacity to repair oxidized base lesions in single stranded DNA, indicating that NEIL3 could occupy a highly specialized role to accurately repair DNA in rapidly proliferating cells. Another study also demonstrated that, similarly to OGG1, siRNA-mediated kd of NEIL3 decreased NSC differentiation ability, resulting in a decrease of both neuronal and astrocytic gene expression after mitogen withdrawal as well as a decrease in the stem cell marker Musashi-1 [37]. Furthermore, a deficiency in NEIL3 led to a decrease in cell proliferation along with an increase in heterochromatin protein 1γ immunoreactivity, a sign of premature senescence, while cell survival remained unaffected. This potentially suggests that OGG1 and NEIL3 play a role in governing essential neural stem cell characteristics.

##### 2.2.3.1. Stroke/Ischemia/Hypoxia

Newborn pigs that were subject to hypoxia and in the following treated by either normothermia or total body cooling showed a significant decrease in transcription of NEIL3 in the hippocampus and cerebellum by hypothermia, but without effect on the accumulation of oxidative DNA damage in genomic DNA [68]. Like for OGG1 and NEIL1, NEIL3 expression in the brain was unaffected by hyperoxia.

Taken together, NEIL3 seems to predominantly play a role in neuronal stem cells and in the proliferation stages of neurons, whereas it rather is down-regulated during later stages of life. Future studies designed to address the role of NEIL3 in neuronal tissue will shed more light on this issue.

### 2.3. The Alkyladenine DNA Glycosylase

The alkyladenine DNA glycosylase (AAG, also named MPG and APNG) is the repair protein that efficiently recognizes and removes different methylated DNA base lesions is [131]. AAG acts on several structurally diverse DNA damages such as 3-methyladenine, hypoxanthine (Hx), ɛA, 7-methyguanine, 1-methylguanine, 1,*N*^2^-ethenoguanine and U [132–136]. Because of the lack of both an alpha-beta fold characteristic to uracil DNA glycosylases (UDGs), as well as a HhH, AAG forms a separate class of DNA glycosylases [7]. Human AAG is present in three different isoforms: A, B and C. The AAG protein levels vary throughout different human tissues, being especially high in the brain, lymph nodes, tonsils, testis and adrenal glands [137]. Strong AAG expression has been reported in the following brain regions: cerebral cortex, hippocampus, lateral ventricle and cerebellum [137].

#### 2.3.1. Involvement of AAG in Neurodegenerative Disorders

AAG mouse models, such as AAG ko and AAG transgenic (AAG-Tg) mice, provided valuable tools to study the influence of AAG-initiated BER on brain development and neurodegeneration [138–141]. Treatment with the alkylating agents methylazoxymethanol (MAM) and methyl methanesulfonate (MMS) induced extreme cerebellar toxicity and dramatically impaired motor function in AAG-Tg mice, while these effects were suppressed in AAG ko animals [131,142]. These findings support the idea that AAG activity, induced by alkylation treatment, promotes accumulation of toxic BER intermediates, while loss of AAG prevents their formation, thus ensuring resistance. Though several lines of evidence strongly indicate that a lack of AAG-initiated BER prevents induction of alkylation induced cell death in different tissues [131,138,142,143], it is important to note that the impact of BER absence on cellular survival largely depends on the type of DNA lesions induced by alkylating treatment, as well as the affected cell type. One such example is the treatment of neuronal and astrocyte cell cultures, obtained from the cerebellum of wt or Aag ko mice, with either chloroacetaldehyde (CAA) or the alkylating agent 3-methyllexitropsin (Me-Lex). Treatment with both CAA and Me-Lex resulted in increased sensitivity of AAG ko neurons, while the sensitivity of AAG ko astrocytes did not differ from the wt cells [144]. Present studies clearly imply an essential and specific role of AAG-mediated BER in alkylation-mediated neurodegeneration.

### 2.4. The Uracil DNA Glycosylases

#### 2.4.1. UNG

The UDG family in eukaryotes can be divided into three subfamilies: the uracil *N*-glycosylase (UNG), the single-strand-specific monofunctional uracil DNA glycosylases (SMUGs) and the mismatch-specific uracil DNA glycosylases (MUGs). Although members of the UDG family have very diverse amino-acid sequences, they share a common alpha-beta fold present in the catalytic active site. Humans and mice have two different UNG isoforms, UNG1 and UNG2 localized in the mitochondria and the nucleus, respectively [145]. UNG protein levels vary in different tissues and cell types. In the human brain UNG levels are (i) extremely high in cerebellar Purkinje cells and neuronal cells of the cerebral cortex; (ii) moderate expression is observed in neuronal cells of the lateral ventricle, and in cerebellar cells of the granular and molecular layer as well as in glial cells; while (iii) very low levels or no UNG is detected in glial cells of both the hippocampus and the lateral ventricle [137]. Several studies clearly demonstrated variations in the activity and levels of UNG with age [25,26,31,146,147]. Neuronal extracts prepared from the cerebral cortex of young (7 days), adult (180 days) and old (720 days) rats showed a dramatic decrease with age in the ability to remove U from the DNA [26]. Supplementation of these extracts with recombinant purified UNG, Pol β and T_4_ DNA ligase significantly restored the loss of BER in aging neurons [26]. Single cell gel electrophoresis experiments of neurons and astrocytes from the cortex of young, adult and old rats revealed a marked increase in the number of UNG sensitive sites with age; further indicating age-dependent decrease in UNG activity [25]. Additionally, analysis of nuclear and mitochondrial UNG activities in different brain regions (the caudate nucleus, frontal cortex, hippocampus, cerebellum and brain stem) of young and adult mice revealed an age-dependent decrease in mitochondrial UNG-initiated BER [146]. In contrast to mitochondria, no region- or age-specific differences were detectable in the UNG nuclear activity, with exception of the cerebellum where uracil incision capacity was reduced with age [146]. Gredilla *et al.* similarly reported reduced UNG1 action in cortical mitochondria, however they did not detect any age-dependent change in the U removal ability in hippocampal mitochondria, while in cerebellar mitochondria UNG1 activity reached its maximum at old age [31]. Taken together, present findings clearly indicate an important role of UNG in different brain regions and suggest that an age-dependent increase in damage to mtDNA might contribute to the normal aging process.

##### 2.4.1.1. Involvement of UNG in Neurodegenerative Disorders

The impact of UNG-mediated repair on mtDNA stability and its role in neurodegeneration were clearly demonstrated through a recent study using a mutated UNG1 (mutUNG1) transgenic mouse model [148]. MutUNG1 removed in addition to U also T from mtDNA, thus promoting mitochondrial instability. Targeted hippocampal expression of mutUNG1 resulted in mtDNA toxicity, decreased mitochondrial respiratory activity, apoptosis, neurodegeneration and impaired behavior [148]. Absence of UNG also strongly influenced mitochondrial stability, with a significant increase in the frequency of the D-1 mtDNA deletion in UNG ko mice [149]. Exposure of UNG ko mice to a folate-deficient diet (FD), a condition frequently associated with stroke, dementia and certain psychiatric disorders, increased mitochondrial mutagenesis in the aged brain and induced a compensatory increase in mtDNA content [149]. Consequences of FD in UNG ko animals were cognitive defects and enhanced mood alterations, such as anxiety and desperation [150]. As a consequence of induced mitochondrial instability and accumulation of DNA damage in general, a lack of UNG in cultured hippocampal neurons directly promoted apoptosis coinciding with p53 up-regulation [151]. This is of particular importance during tissue repair after brain ischemia, where a major increase in infarct size was observed in UNG ko mice when compared to wt animals [152]. Besides its impact on neuronal survival and brain integrity in general, several findings clearly suggested an important role of UNG in neurodegenerative disorders. Total protein analysis of temporal lobe autopsies from four tauopathies indicated a significant change in UNG protein levels [153]. Further, both the activity of UNG-mediated BER as well as UNG protein levels were decreased in the inferior parietal lobule (IPL) of 10 sporadic AD patients [154]. Changes in BER capacity were not only detectable in disease-affected regions such as the IPL, but were also present in unaffected regions like the cerebellum [154]. Interestingly, while total BER capacity decreased with age in the brain of healthy individuals as expected, most AD patients had low BER levels independently of their age [154]. Impaired UNG-mediated BER was also detected in brains of amnestic mild cognitive impaired patients and this defect correlated with the abundance of neurofibrillary tangles [154]. Current studies suggested that the balance of UNG-mediated BER is potentially important to prevent premature aging and the onset of neurodegenerative disorders.

#### 2.4.2. TDG

The thymine DNA glycosylase (TDG) is a member of the MUG subfamily. Besides T, TDG recognizes and excises U, 5-FU, ɛC, 5-hydroxymethyluracil, 5-formylcytosine and 5-carboxylcytosine when base paired with G [155–163]. TDG ko in mice is embryonically lethal, suggesting an essential function of TDG during development [164,165]. While the expression of TDG in human brain is documented in detail, studies in rat brains indicated that similar to UNG, TDG levels were inversely correlated with age [166,167]. Future studies are needed to reveal a role of this unique glycosylase in brain integrity and consequentially in neurodegeneration.

So far not much is known about the role of the remaining two members of the UDG family in brain development and homeostasis.

## 3. BER Proteins other than DNA Glycosylases

### 3.1. Apurinic/Apyrimidinic Endonuclease 1

APE1 is a multifunctional enzyme with a pivotal role in BER, by processing AP sites, and in the regulation of transcriptional activity by redox activation of transcription factors (such as Fos and Jun) ([168–176], and reviewed in [177]). Depletion of APE1 in cultured hippocampal and sensory neurons sensitized the cells markedly to oxidative DNA damage induced by H_2_O_2_, reflected in reduced cell viability, increased caspase-3 activity and histone H2AX phosphorylation (γH2AX) [178]. In contrast to depletion, APE1 overexpression was neuroprotective in dorsal root ganglion neurons exposed to cisplatin [179]. In addition, it has been shown that it is the DNA repair function of APE1 that is crucial for cell survival of post-mitotic cells exposed to oxidative stress [180]. Determining the APE1 activity in cortical astrocytic and neuronal extracts derived from young (7 days), adult (6 months) and old (2 years) rats revealed an age-dependent decrease in the activity in adult compared to the young animals [25]. This reduction remained with age and was therefore also apparent in old rats. Exposing young (3 months) and aged (30 months) rats to 100% oxygen, Edwards *et al.* showed in young animals a reflective increase of APE1 protein levels in the hippocampus and basal forebrain, whereas no significant changes were detected in aged rats, suggesting an impaired responsiveness to oxidative stress [181].

#### 3.1.1. Alzheimer’s Disease

A study of the APE1 hippocampal expression in human AD brains revealed elevated APE1 levels both in senile plaques, a histopathological hallmark of AD, and in injured neurons [182]. This increase was further found to be localized to the nuclear fractions of AD brains [183], which was confirmed in a immunohistochemical analysis of the cerebral cortex, where an intensive nuclear APE1 signal in all cortical layers was detected [184]. Aβ, a major contributor to AD development, is known to induce oxidative stress in neurons [185]. Tan *et al.* investigated the impact of various Aβ concentrations on APE1 levels and cell survival in isolated rat hippocampal neurons [186]. Interestingly, treatment with high concentrations of Aβ (5 μM) caused a reduction in cellular APE1 levels and activity, which correlated with extensive neuronal degeneration of the cultured hippocampal neurons. In contrast, lower concentrations of Aβ (1 μM) induced APE1 expression and activity, resulting in no substantial loss of the neurons [186]. The cyclin-dependent kinase 5 (Cdk5) was shown to regulate APE1 through phosphorylation, leading to a reduction of its endonuclease activity [187]. Subsequent accumulation of DNA damage, together with the finding that levels of phosphorylated APE1 were increased in brain tissue from AD and PD patients, might implicate Cdk5-mediated APE1 phosphorylation in the development of these neurodegenerative disorders. Independently of the endonuclease activity, but through its redox function, APE1 was found to mediate neuroprotection against Aβ and H_2_O_2_ via induction of the glial cell-derived neurotropic factor (GDNF) receptor α1 transcription, thereby increasing the GDNF responsiveness [188]. In a very recent proteomic study, where neuronal cells were challenged with an Aβ peptide fragment (25–35), novel interaction partners of APE1 were identified [189]. Among them, (i) tropomodulin 3, involved in the synaptic activity; (ii) heterogeneous nuclear ribonucleoprotein-H1, a regulator of alternative splicing and (iii) the pyruvate kinase 3 isoform 2, a key enzyme in the glycolysis; all of these factors might have a functional relevance for neuronal cell survival and Aβ resistance. In addition, a potential association between the APE1-D148E SNP and the onset of AD was investigated, however no significant correlation was found [190].

#### 3.1.2. Involvement of APE1 in other Neurodegenerative Disorders

Decreased APE1 levels were found in patient cells affected by Ataxia with Oculomotor Apraxia Type 1 (AOA1) [191], a neurodegenerative disorder originating in mutations of the *APTX* gene [192,193], which results in a cellular aprataxin deficiency [194]. Comparable findings were also obtained in ALS patients, where frontal cortical APE1 levels, as well as activity were significantly reduced [195], and in some cases missense mutations within the *APE* gene were identified [196]. ALS manifests in the progressive loss of motor neurons [197] and appears in a sporadic as well as a familial form [198]. For the sporadic form, a significant association with the D148E APE1 polymorphism was shown [199]. In contrast to the analysis of frontal cortical levels, a study by Shaikh *et al.* indicated increased APE1 levels in the spinal cord and motor cortex of ALS patients and showed that protein extracts from this tissue samples were more proficient in *in vitro* processing of AP sites [200]. Hyperactivity of APE1 potentially also contributes to the genomic instability by resulting in an increased number of extremely harmful DNA breaks.

In a rat model where epileptic-like seizures were induced by the application of kainic acid (KA), a subsequent induction of APE1 expression was observed in KA-vulnerable brain regions (CA1, CA3 and hilar subregions of hippocampus, pyriform cortex, amygdala and thalamus) [201]. Furthermore, APE1 colocalized with the BER protein XRCC1, the oxidative DNA damage marker 8-oxo-G, the tumor suppressor p53 and also with fragmented DNA, as assessed by TUNEL staining [201]. These findings thus indicate that BER is activated but not sufficient to counteract excitotoxicity-mediated neuronal cell death.

#### 3.1.3. Stroke/Ischemia/Hypoxia

A cold injury-induced brain trauma (CIBT) mouse model revealed an early post-traumatic decrease of APE1 levels within the lesion, which preceded later DNA fragmentation [202]. Similar observations were made after severe traumatic cortical brain injury [203]. However, the outer boundary area that survived CIBT showed a significant increase in APE1 immunoreactivity [202]. Transient focal cerebral ischemia (FCI) [204] or a defined hypoxic-ischemic insult [205] resulted in decreased APE1 protein levels, a reduction exclusively detected in the hippocampus. In addition, APE1 levels selectively decreased in the hippocampal CA1 neurons 2 days after transient global cerebral ischemia (GCI), which was followed by DNA fragmentation after 3 days [206]. Intra cerebral application of the pituitary adenylate cyclase-activating polypeptide (PACAP) in the context of transient GCI reversed the effect, by inducing APE1 expression in hippocampal CA1 neurons, which correlated with improved cell survival [207]. This neuroprotective effect of PACAP was dependent on the DNA repair activity of APE1, as was shown through a loss-of-function rescue attempt of APE1 deficient cells, by overexpressing DNA repair-incompetent APE1. Upon transient spinal cord ischemia, spinal APE1 levels decreased while oxidative DNA damage increased [208]. Interestingly, an ischemic tolerance could be established by sub-lethal ischemic preconditioning, which resulted in subsequent up-regulation of APE1 levels and other BER proteins and therefore better neuroprotection in the case of severe ischemia [63]. On the other hand, APE1 overexpression was shown to increase cell viability of cultured hippocampal and sensory neurons after ionizing radiation-induced DNA damage [209]. Glutamate-induced oxidative DNA damage was found to cause an increase in APE1 expression in rat cerebral cortical neurons via a pathway involving the cAMP-response element-binding protein, thereby improving the DNA repair activity of oxidized lesions [210].

In summary, the multifunctional enzyme APE1 is implicated in a broad spectrum of neuropathologies via both, its endonuclease and redox activity. However, the exact regulation of APE1 in this context and the underlying mechanisms remain to be investigated.

### 3.2. Polynucleotide Kinase

The polynucleotide kinase (PNK) is a bifunctional enzyme exhibiting a 5′-DNA kinase and a 3′-phosphatase activity [211]. The removal of 3′-phosphate groups renders DNA ends accessible for Pols, an important step for promoting BER upon base excision by either NEIL1, NEIL2 or NEIL3 (reviewed in [212]). Highest expression in human tissues of PNK was observed in the spleen, testis, heart and pancreas, whereas brain levels were rather low [211,213]. PNK is known to interact during DNA repair with the scaffold protein XRCC1 [214]. Disruption of this interaction impairs the DNA repair capacity following oxidative stress [215]. In order to rescue this phenotype, overexpression of 3′-phosphatase-proficient PNK was needed, indicating the 3′-phosphatse activity to be critical for efficient repair of oxidative DNA damage.

#### 3.2.1. Involvement of PNK in Neurodegenerative Disorders

A recent study by Shen *et al.* linked four mutations in the *PNKP* gene to an autosomal recessive disease characterized by microcephaly, early-onset, severe seizures and developmental delay (MCSZ) [216]. The mutations were either frame-shift mutations (T424Gfs48X and exon15Δfs4X) due to small deletions or duplications within the kinase domain, resulting in truncated PNK proteins or point mutations (L176F and E326K) within the phosphatase domain. Assessing the DNA repair capacity by comet assay in patient-derived lymphocytes after H_2_O_2_ or camptothecin treatment, revealed a significant impairment in the case of MCSZ cells compare to healthy controls and in addition, PNK protein levels were found reduced in these patient cells[216]. Interestingly, a further study showed *in vitro* that PNK^T424Gfs48X^, PNK^exon15Δfs4X^ and PNK^L176F^ mutant proteins exhibit a markedly decreased 5′-DNA kinase activity [217]. However, a moderate reduction in the 3′-DNA phosphatase activity was only observed in the case of recombinant PNK^L176F^. Analysis of phosphatase and kinase activities in MCSZ patient cells with decreased mutant PNK levels [216], revealed an overall marked reduction [2,17]. Furthermore, alkaline comet assay analysis of cells from affected individuals exhibiting the PNK^T424Gfs48X^ and PNK^exon15Δfs4X^ mutations as well as of cells homozygous for PNK^E326K^, revealed inefficient repair of DNA strand breaks upon γ-irradiation, whereas camptothecin treatment only led to an accumulation of DNA damage in cells containing both, mutated PNK^T424Gfs48X^ and PNK^exon15Δfs4X^[217]. Even though PNK appears to be crucial for proper neurodevelopment, further mechanistical studies will be needed to unravel the potential link between impaired DNA repair capacity and the development of MCSZ.

### 3.3. DNA Polymerase β

The DNA repair enzyme Pol β belongs to the X family of DNA Pols. In addition to the polymerase activity, exhibited particularly on short gaps, it also possesses a dRPlyase activity and associates with BER proteins such as XRCC1 and DNA ligase III [218–235]. Thereby, Pol β is considered to be the major BER Pol (reviewed in [236]). It is constitutively expressed in most tissues, with the highest levels found in testis and brain [237]. The generation of a Pol β ko mouse model revealed that, besides growth retardation and insufficient lung ventilation leading to immediate postnatal death, these mice also displayed altered neurogenesis [238]. The defect in neurogenesis was reflected in vast apoptotic cell death in the developing cortex, hindbrain and dorsal root ganglion. Additional ko of p53 in Pol β deficient mice (Pol β ko and p53 ko) abolished the neuronal cell death, thus proving the p53-dependency of this apoptotic pathway [239]. However, neonatal lethality remained even in the absence of p53. Ko of the DNA-dependent protein kinase catalytic subunit (DNA-PKcs), an enzyme involved in the non-homologous end-joining (NHEJ), in a Pol β deficient background exhibited even more pronounced growth retardation and neuronal apoptosis as well as earlier lethality compared to Pol β ko [240].

The fact that Pol β activity changes with aging was demonstrated in a study, where incubation of rat neuronal extracts from young, adult and old animals with synthetic 1 and 4 nucleotide gap constructs resulted in reduced BER capacity with age [241]. Though the gap repair activity was markedly impaired with age, complementation with rat liver Pol β rescued the activity. A further study by Cabelof *et al.* confirmed these findings by showing that Pol β activity, protein and mRNA levels decreased significantly in brain tissue with age by comparing young (4 months) and old (24 months) C57BL/6 mice [242]. The biological relevance of this age-associated decrease in Pol β was further underlined by the observed increased mutation frequency in old animals. Interestingly, caloric restriction was able to largely reverse this age-dependent decline [243].

#### 3.3.1. Alzheimer’s and Parkinson Disease

AD cell culture models demonstrated that the application of Aβ to cortical neurons induces cell cycle reentrance followed by DNA replication resulting in reflective apoptosis of these cells [244]. Pol β was shown to be a leading Pol mediating *de novo* DNA synthesis after Aβ-induced cell cycle reentrance and thereby to play a role in the neuronal loss [245]. Consequently, in Aβ-treated cortical neurons, Pol β was also found to co-immunoprecipitate with cell division cycle 45 (Cdc45) and the DNA primase in nucleoprotein fragments, implicating its association with the DNA replication fork [246]. Aβ treatment of neural progenitor cells induced Pol β expression, with the consequence of cell differentiation along the neuronal lineage [247]. However, Weissman *et al.* showed overall decreased Pol β protein levels in AD brain tissue accompanied by reduced single nucleotide gap-filling activity [154]. Cell cycle re-entry was also observed in cultured cerebellar granule cells treated with the neurotoxin 1-methyl-4-phenylpyridinium (MPP^+^), which is known to mimic PD by selective toxicity against dopaminergic neurons [248]. MPP^+^-mediated cell death was accompanied by increased Pol β expression. Interestingly, reduction of Pol β activity by either inhibition with dideoxycitidine or the expression of a dominant negative Pol β variant in these cells attenuated the neuronal loss [248].

#### 3.3.2. Triplet Repeat Expansion Diseases

Several studies addressed the role of Pol β in the CAG triplet repeat expansion associated with HD. Kovtun *et al.* showed in an *in vitro* OGG1-initiated BER assay, that Pol β tends to perform strand displacement DNA synthesis within CAG repeats instead of single-nucleotide incorporation, resulting in longer DNA products [53]. Thus indicating that the initiation of BER by OGG1 in a CAG sequence contributes to the TNR expansion by Pol β-mediated strand displacement. Supporting this finding, a further study showed that multinucleotide incorporation by Pol β results in strand displacement and the formation of CAG hairpins that become stabilized and promote repeat expansion [249]. Pol β was also shown to accumulate along CAG repeats in the striatum of HD mice, the brain region most susceptible to degeneration in HD patients (for more details see Chapter 3.6.) [57]. Furthermore, Goula *et al.* recently determined the protein levels of the major BER proteins in the striatum, as well as the HD-spared cerebellum of HD transgenic mice [250]. While it was previously shown that Pol β protein levels were not significantly changed in these tissues, DNA ligase I, FEN1, APE1 and XRCC1 levels were increased by at least 2-fold in cerebellum [57,250]. In addition, *in vitro* repair of AP-sites with either (i) purified BER proteins in the striatum-specific stoichiometry or (ii) with wt and HD mice striatum extracts, was shown to be less proficient than cerebellar repair regardless of the sequence context [250]. The same study demonstrated that lesions within CAG repeats tend to be repaired via LP-BER and that this process is more efficient under cerebellar conditions compared to the striatal ones. Moreover, a lesion closer to the 3′ end of the repeat sequence was more efficiently repaired than a 5′ lesion, most probably due to the fact that the formation of stable hairpins becomes impaired the closer a lesion is to the 3′ end of the repeat tract [250]. Taken together these findings suggest a potentially important role of Pol β mediated repair in the onset of triplet repeat expansion diseases, such as HD.

#### 3.3.3. Stroke/Ischemia/Hypoxia

Pol β activity was observed to be up-regulated in cerebral cortical neurons of newborn piglets exposed to hypoxia, and this was suggested to be beneficial to reduce hypoxia-induced DNA damage [251]. Upon transient FCI in rats, an increase in markers for oxidative DNA damage, namely 8-oxo-G and AP sites could be detected [252]. These lesions were efficiently repaired during reperfusion in the surviving cortex, which was proposed to be at least partially due to a protective long-lasting up-regulation of Pol β expression as well as its activity. As already described for APE1, ischemic preconditioning also elevated the protein levels of Pol β as well as XRCC1 and DNA ligase III [253]. This up-regulation in particular of SP-BER proteins was implicated to play a pivotal role in the neuroprotection observed in subsequent severe ischemic episodes.

Summarizing, Pol β was shown to be crucial for proper neurodevelopment, whereas later on Pol β levels decline in an age-dependent manner. In the context with AD, spurious de novo DNA synthesis contributes to neuronal loss. However, upon hypoxic insults, Pol β activity mediates neuroprotection.

### 3.4. DNA Polymerases δ and ɛ

The Pols δ and ɛ belong together with Pol α to the B-family of DNA Pols and are the main Pols involved in lagging and leading strand replication [254–257]. Due to this function as replicative Pols, their DNA synthesis accuracy is high on undamaged DNA templates (reviewed in [258]). Besides DNA replication, the role of Pol δ and ɛ in LP-BER is supposed to be the accurate elongation after repair synthesis initiated by Pol β [259].

Even though Pol β was for a long time assumed to be the only Pol present in the brain, Pol δ and ɛ activities were reported in developing as well as aging rat cerebral cortical neurons [260]. Analyzing TNR expansions in *Saccharomyces cerevisiae* revealed an involvement of yeast Pol δ together with the DNA helicase Srs2 in blocking TNR expansion [261]. Also, Pol δ has been found together with the DNA helicase Werner syndrome protein (WRN) to be important in resolving TNR-based hairpin structures in HeLa nuclear fractions complemented with recombinant proteins [262].

### 3.5. X-Ray Repair Cross Complementing 1 Protein

The XRCC1 protein represents a crucial scaffold protein in the BER [222,263]. Among other roles, its interaction with the Pol β and DNA ligase III contributes to the 1nt-gap filling reaction and subsequent DNA ligation especially in the context of SP-BER ([227,264–266], and reviewed in [267]).

In 1999, Fang-Kircher *et al.* analyzed for the first time mRNA levels of different DNA repair proteins in human brain tissue of deceased Down’s syndrome patients [268]. Interestingly, a significant up-regulation of XRCC1 mRNA was found in the temporal, parietal and occipital lobes of these patients. Increased XRCC1 expression might be explained by the higher levels of ROS detected in Down’s syndrome neurons [269]. However, frontal lobe and cerebellar levels of XRCC1 were equal or even significantly lower in comparison to those observed in control brains [268].

That XRCC1 is particularly important in differentiated neurons for the repair of oxidative DNA damage has been shown in differentiated human SH-SY5Y neuroblastoma cells [270]. SH-SY5Y XRCC1 kd cells displayed lower survival upon treatment with the oxidizing agents menadione or paraquat compared to control cells. No differences between proficient and deficient backgrounds were detected in dividing (non-differentiated) SH-SY5Y cells. In contrast, treatment of dividing SH-SY5Y XRCC1 kd cells with Hx, an additional inducer of oxidative damage by generating extracellular H_2_O_2_, resulted in increased sensitivity [270]. Taken together, these results support the general idea that requirements for the DNA repair proteins largely depend on the type of oxidative damaged that is induced. Consistent with these findings, XRCC1 heterozygous primary mouse cerebellar granule cells as well as XRCC1 kd human fetal brain neurons displayed a higher sensitivity towards menadione treatment [270].

Isolated granule cells from a neuronal-specific XRCC1 ko mouse model (XRCC1^Nes−Cre^) exhibited impaired DNA repair capacity after exposure to H_2_O_2_, as assessed by comet assay [271]. Furthermore, neurons in different brain regions of XRCC1^Nes−Cre^ mice accumulated DNA damage in an age-dependent manner, shown by increased amounts of persistent γH2AX foci, a known marker of DNA double-strand breaks (DSB) and most likely DNA single-strand breaks (SSB). Interestingly, histological analysis of the cerebellum revealed a marked p53-dependent loss of basket, stellate and Golgi interneurons, all of which are important for fine-tuning of the cerebellar output [271]. Furthermore, accumulation of γH2AX foci associated with gliosis and increased c-Fos staining within distinct hippocampal regions indicated altered hippocampal homeostasis [271]. These features closely resemble the neuropathological characteristics found in temporal lobe epilepsy [272]. In a rat model where epileptic-like seizures were induced by KA, an induction of XRCC1 was observed after 16 h of seizure-onset in KA-vulnerable brain regions (CA1, CA3 and hilar subregions of hippocampus, pyriform cortex, amygdala and thalamus) [201]. As KA is known to cause oxidative stress [273], up-regulation of XRCC1 was thought to happen as a compensatory mechanism for the increased levels of DNA damage.

#### 3.5.1. Alzheimer’s and Parkinson Disease

The discovery of XRCC1 polymorphisms gave raise to studies investigating a potential predisposition to diseases [274]. AD belongs to the most common diagnosed age-related neurodegenerative disorders resulting in dementia and it is often histologically characterized by the presence of Aβ plaques, neurofibrillary tangles and severe neuronal loss (reviewed in [275]). Since the R194W XRCC1 polymorphism lies within a conserved amino acid residue sequence [274], its potential functional relevance for neurodegeneration was addressed in a case-control study focusing on the late-onset AD in a Turkish population [276]. An increased risk for late-onset AD in the presence of the R194W polymorphism was proposed [276]. However, the risk estimates did not reach a statistically significant level and were not confirmed in a later comparable study among Han Chinese people [277]. It therefore remains under debate, whether the R194W XRCC1 polymorphism promotes the onset of AD. The analysis of two additional XRCC1 polymorphisms, namely R280H and R399Q revealed no dependency between allele frequency and the onset of AD [190]. However, an increased risk was found for the development of PD in the presence of XRCC1 R399Q [278].

#### 3.5.2. Stroke/Ischemia/Hypoxia

XRCC1 is not only implicated in chronic neurodegeneration, but also in neuronal loss after acute central nervous system injuries, including the cerebral ischemia-reperfusion phenomenon [279]. It is well established, that brain ischemia/reperfusion, a condition that resembles a stroke, goes along with an excessive production of ROS (reviewed [280,281]). In mice subjected to transient FCI by occlusion of the MCA, it was shown that XRCC1 levels decreased shortly after reperfusion and remained low until 24 h in the total MCA territory [279]. The loss of XRCC1 coincided with a positive TUNEL staining, corresponding to fragmented DNA arising 24 h later. A CIBT mouse model further supported this observation, where a similar early decline in XRCC1 levels within the injured region preceded later DNA fragmentation [282]. Thus, these studies indicated a potential role of the early XRCC1 decrease in the DNA damage-mediated neuronal cell death in traumatic brain regions [279,282].

Taken together, the scaffold protein XRCC1 was shown to be particularly important for DNA repair in post-mitotic neurons. Further association with the Down’s syndrome, seizure episodes and ischemic insults render XRCC1 a critical player in CNS homeostasis.

### 3.6. Flap Endonuclease 1

The multifunctional enzyme FEN1 possesses a 5′→3′-exonuclease and a 5′-endonuclease activity [283–288]. The latter function is of special importance in the context of LP-BER, as FEN1 removes the arising 5′-flap structures after strand displacement DNA synthesis, thereby allowing subsequent DNA ligation (reviewed in [289]).

#### 3.6.1. Triplet Repeat Expansion Diseases

In the severe neurodegenerative disorder HD, BER initiated by OGG1 has already been implicated to contribute to the CAG trinucleotide expansion in somatic cells, as discussed above [53]. In addition, a study in HD mice showed, that oxidative damage specifically accumulates along CAG repeats in a length-dependent manner [57]. This event is specifically taking place in the striatum, the brain region most prone to degeneration in HD patients, when compared to the disease-spared cerebellum. Even though gap-filling activity is reduced in the striatum several factors, such as: (i) pronounced accumulation of Pol β at CAG repeats; (ii) promoted Pol β-mediated strand displacement activity; and (iii) low 5′-flap endonuclease activity by FEN1, contribute to the somatic instability in the context of LP-BER [57]. This is further in line with model of HD which suggests that the Pol β generated displaced strand, when not efficiently removed by FEN1, forms a hairpin structure that can become stably integrated leading to trinucleotide expansion. The stoichiometry between BER proteins seems to be an important factor for tissue-specific trinucleotide expansion-vulnerability [57]. Elevated levels of FEN1 in the cerebellum were implicated to significantly contribute to the increased BER efficiency on CAG substrates observed by measuring the repair capacity with BER proteins in the cerebellar stoichiometry (discussed in more detail under 3.3.) [57,250]. This effect was not observed when BER efficiency was addressed under the striatum-specific conditions [250]. Liu *et al.* similarly reported that FEN1 might facilitate TNR expansion, however through a slightly different mechanism by alternate cleavage of hairpin structures arising during Pol β multinucleotide DNA synthesis and strand displacement during LP-BER [249]. As FEN1 is unable to process 3′ ends of stable hairpins, it rather tends to cleave lose 5′-flaps of the hairpin, thereby providing ligatable nicks and potential TNR expansion. A comprehensive overview of the FEN1 involvement in the TNR expansion is presented in a recent review by Liu and Wilson [290].

### 3.7. DNA Ligase I/III

The DNA ligase I in association with PCNA is targeted to the replication machinery where it is important for Okazaki fragment joining [291]. In the context of BER it acts in the final step of LP-BER by sealing nicked DNA (reviewed in [292]). The DNA ligase III, on the other hand, is mainly implicated in DNA ligation during SP-BER where it is in complex with the scaffold protein XRCC1 [265,266]. Both, the DNA ligase I and III are shown to be essential for proper embryonic development, as studied in mice (reviewed in [292]). Moderate expression of the DNA ligase I was found in the cerebellum, lateral ventricle and cerebral cortex, whereas levels in the hippocampus were rather low [137]. However, in comparison to cerebellar levels, DNA ligase I is markedly reduced in the striatum where it might be involved in the observed less efficient BER of lesions within CAG repeats, thus potentially contributing to TNR instability [250]. High levels of DNA ligase III were observed in the cerebellum and the cerebral cortex decreasing to moderate levels in the hippocampus and lateral ventricle [137]. A DNA ligase I deficiency described in a female patient was accompanied with continuous infections due to a compromised immune response, sensitivity towards sunlight, growth retardation and delayed development [292]. The neurodegenerative disorder spinocerebellar ataxia with axonal neuropathy-1 (SCAN1) originates from mutated tyrosyl phosphodiesterase 1 (TDP1), a protein involved in the repair of DNA SSB [293]. Interestingly, the DNA ligase III was found to directly interact with mutated TDP1 in SCAN1, forming a catalytically inactive complex, thereby potentially contributing to a defective SSB repair [294]. A further link between DNA ligases and neurodegeneration is given in the neurological disorder AOA1, where aprataxin, a protein important for the removal of adenylate groups at single-strand nicks [295], is mutated [192,193]. Therefore, deficiency in the processing of adenylate groups by aprataxin results in abortive ligation attempts by the DNA ligase III, which might result in the accumulation of DNA SSB [295].

#### 3.7.1. Stroke/Ischemia/Hypoxia

As already observed for other BER proteins, ischemic preconditioning resulted in an induction of the DNA ligase III in neuronal and glial cells [253]. This up-regulation of the BER pathways was further reflected in the increased BER activity in nuclear brain extracts from preconditioned animals [253].

In summary, even though not much is known so far about DNA ligases in the physiology and pathology of the CNS, initial hints point towards an involvement in distinct neurodegenerative disorders.

## 4. Conclusions and Future Perspectives

As a consequence of high oxygen metabolism, an efficient BER pathway is needed to ensure genomic stability and brain homeostasis. The majority of BER proteins are highly expressed in the brain, however present data clearly indicate that the expression pattern is not homogenous and that it differs from one brain region to another. In addition, levels of BER proteins change with age, resulting in accumulation of DNA lesions and genomic instability. Tissue-specific and age-dependent expression of major BER proteins suggests the existence of a very complex and highly regulated DDR in the CNS. This complexity is most probably also a reason why studies addressing the role of BER proteins in brain physiology and pathology, using various different models, resulted in different and sometimes contradictory observations. However, changes in BER protein levels and the DNA repair capacity have been correlated with some of the most common neurodegenerative disorders (Table 1), thus indicating the importance of understanding the mechanisms that ensure tight regulation of BER protein expression and activity. Though numerous studies compared the BER status between different brain regions during development, as well as between tissues of healthy individuals and patients suffering from various neurodegenerative disorders; still very little is known about the means by which BER is regulated in the brain. By revealing pathways important for balancing BER, we might be able to understand how changes in the BER capacity during lifetime, as well as neurodegeneration, contribute to aging and disease onset/progression, respectively.

## Figures and Tables

**Table 1:** Overview of BER proteins and their involvement in physiology and pathology of the brain. The information listed is an overview of the data presented in the manuscript text. ↑ stands for up-regulation or an increase, while ↓ signifies either down-regulation or decrease.

Protein			Physiological expression in brain	Expression changes induced by		Changes associated with neurodgenerative disorders		Brain specific effect of knockout/knockdown
	Protein family							
**DNA glycosylases**	Helix-hairpin-helix family	**OGG1**	- ↓ postnatal - ↑ from 8 weeks - ↓ age-dependently	↑	- cigarette smoke - dieldrin-proliferating cells - SIF in murine brains	PD	- ↑	- differentiation shiftneural to astrocytic lineage - mild PD phenotype with age - ↑ sensitivity to dopaminergic substances and ischemia-induced DNA damage - combination with CSB kd—no effect on CS phenotype
						ALS	- S326C increased risk - ↑ in presymptomatic SOD1 mice	
				↓	- dieldrin-differentiated cells - fenvalerate	HD	- OGG1 increases TNR instability, especially the S326C - ↓ in striatum of HD mice	
				No change	- lead (Pb)	Stroke/Ischemia	- various effects depending on the model used	
						AD	- ↑ but also ↓ observed	
		**MUTYH**	- ↑ in neonate and adult brain - ↓ with age	None reported		PD	- ↑	None reported
						Stroke/Ischemia	- mainly ↑	
						Other disorders	- possibly ↓ in equine cerebellar abiotrophy	
		**MBD4**	None reported	None reported		Diverse disorders	- ↑ in schizophrenia and bipolar disorder patients	None reported
		**NTHL1**	None reported	None reported		Diverse disorders	- no association with MS risk	None reported
	Endonuclease VIII-like	**NEIL1**	- ↑ mid-age, during differentiation - ↓ with age - minor changes in hippocampal mitochondria over lifespan	None reported		Stroke/Ischemia	- no changes by OGD in hippocampal slice cultures, ↓ by hypothermia	- impaired memory and increased brain damage after ischemia/reperfusion in ko mice
		**NEIL2**	- ↑ during differentiation	None reported		Stroke/Ischemia	- no changes after OGD	None reported
		**NEIL3**	- stem cell rich regions, also in early embryos - ↓ with age	None reported		Stroke/Ischemia	- ↓ in hypoxia	- ko with ↓ neuronal progenitors and NSC differentiation ability
		**AAG**	- highly expressed in several brain regions	None reported		None reported		- ko results in suppression, while Tg in increase of toxicity induced by alkylating agents
	UDG	**UNG**	- varying expression depending on brain region and age	None reported		AD/TNR disorders	- changed in tauopathies and ↓ in AD patients	- ko and Tg with neurodegeneration - ko ↑ ischemic infarct size
		**TDG**	None reported	None reported		None reported		- ko embryonic lethal
**Endonucleases**		**APE1**	- ↓ with age	↑	- 100% O_2_ in brains of young rats, but not in old ones	AD	- ↑ levels in patients, varying expression upon Aβ treatment - ↑ levels of p-APE1 (less active) - no significant correlation with D148E	None reported
						PD	- ↑ levels of p-APE1 (less active)	
						HD	- 2-fold increase in cerebellum HD mice	
						Stroke/ischemia	- ↓ in several models of hypoxia, hypothermia, stroke and trauma	
						Other diseases	- ↓ in AOA patients - both ↑ and ↓ in ALS patients detected - association of missense mutations, D148E - ↑ in epilepsia model	
		**FEN1**	None reported	None reported		HD	- implicated in TNR expansion, increased in cerebellum of HD mice	None reported
		**PNK**	- low expression	None reported		MCSZ	- multiple mutations associated	None reported
**DNA polymerases**		**Pol β**	- constitutive expression - ↓ activity with age	None reported		AD	- Aβ induced Pol β-mediated cell cycle reentrance, neuronal loss and differentiation of neural progenitors to neuronal lineage - MPP + induces Pol ββ-mediated cell cycle reentrance and cell death	- neonatal lethal, altered neurogenesis in ko mice, which is p53 dependent and more pronounced in a DNA-PKcs ko background
						HD	- Pol β accumulation along CAG repeats in striatum of HD mice	
						Stroke/ischemia	- ↑ in several models	
		**Pol δ + Pol ε**	None reported	None reported		HD	- Pol δ blocks TNR expansion together with Srs2 and resolves srs1 and resolves TNR-based hairpin structures together with WRN	None reported
**Scaffolding**		**XRCC1**	None reported	None reported		AD	- R194W and R399 ↑ risk, no effect by R280H/R399Q	- XRCC1^nes−cre^ ko mice age-dependent accumulation of DNA damage, loss of certain neurons in the cerebellum and altered hippocampal homeostasis
						HD	- 2-fold increase in cerebellum HD mice	
						Stroke/ischemia	- ↓ in several models of ischemia, hypothermia	
						Other diseases	- ↑ levels in some parts of the brains of Down’s syndrome patients, and ↓ in others - ↑ levels in a rat epilepsia model	
**DNA ligases**		**DNA ligase I**	- moderate in cerebellum, lateral ventricle and cerebral cortex - ↓ in hippocampus and striatum			HD	- 2-fold ↑ in cerebellum HD mice	- essential for embryonic development
		**DNA ligase III**	- ↑ in cerebellum and cerebral cortex - moderate in hippocampus and lateral ventricle			SCAN	- association due to interaction with TDP1?	- essential for embryonic development
						AOA1	- association?	
